# Relations of the Spiritual Well-Being Questionnaire Domains with Other Areas of Well-Being: A Commentary Based on Existing Published Studies

**DOI:** 10.1007/s10943-025-02410-7

**Published:** 2025-08-06

**Authors:** Rapson Gomez

**Affiliations:** 1 Institute of Health and Wellbeing, Federation University Australia, Ballarat, VIC Australia; 2 Applied Health, School of Health and Biomedical Science, RMIT University, Melbourne, VIC Australia

**Keywords:** Spiritual well-being questionnaire, SWBQ, General well-being, Spiritual health and life-orientation measure, SHALOM

## Abstract

The Spiritual Well-Being Questionnaire (SWBQ) measures the spiritual well-being domains of personal, communal, environmental, and transcendental well-being as proposed in Fisher’s four-factor model of spiritual well-being. This commentary provides a critical overview of the literature on the associations of the SWBQ domains of spiritual well-being with other areas of general well-being. The commentary revealed consistent positive associations for the personal domain and, to a lesser degree, for the communal domain, with other areas of well-being. The associations for the environmental domain were rare, and the associations were often absent for the transcendental domain. On the basis of observations reported here, the personal domain in the SWBQ has the best concurrent/external validity in terms of relation with some aspects of general well-being, whereas the other domains have limited relevance.

## Introduction

Spirituality remains difficult to precisely define, but there is general agreement that it refers to a connection with a larger reality that gives one’s life meaning, experienced through a religious tradition or, increasingly in secular Western culture, through meditation, nature, or art (Garssen et al., 2016). Related to spirituality, spiritual well-being is viewed as reflecting a sense of purpose, peace, and fulfillment in that connection (Ellison, 1983; Ghaderi et al., 2018; Ryff, 1989). Existing literature indicates that spiritual well-being has been closely associated with other areas of wellbeing, such as physical, emotional, psychological, social, and health (Ellison, 1983; Ghaderi et al., 2018; Moberg, 1979; Paloutzian & Ellison, 1982; Ryff et al., 1989; Zarzycka et al., 2024). In this commentary, these other areas of well-being are referred to collectively as “general well-being”.

Traditionally, the term ‘spirituality’ has been used to refer to an individual’s search for connections with a greater power, such as God (Joseph et al., 2017). More recently, spirituality has been understood more broadly as an attempt to discover meaning and significance in various aspects of life (Woods & Ironson, 1999). For example, the National Interfaith Coalition on Aging (NICA, 1975) defines spirituality as the affirmation of life through relationships with oneself (personal, i.e., beliefs and perceptions about one’s own existence), others (communal, i.e., relatedness with other people and the community), nature (environmental, i.e., relationships with the environment and nature), and the transcendental (i.e., beliefs and deep connections with a higher power, including God).

In line with NICA’s conceptualization of spirituality, Fisher (1998) proposed a multidimensional model in which spiritualty and spiritual well-being are understood as reflecting the extent to which individuals live in harmony with their relationships with oneself (personal), others (communal), the environment (nature), and God (or transcendental entity). Building on the NICA (1975) framework and its elaboration by Fisher (1998), Gomez and Fisher (2003) published the Spiritual Well-Being Questionnaire (SWBQ) to measure the personal, communal, environmental, and transcendental dimensions of spirituality and spiritual well-being.

### The Spiritual Well-Being Questionnaire (SWBQ)

The SWBQ consists of 20 items, with five items corresponding to each of the spiritual domain. The proposed theoretical model for SWBQ follows a four-factor oblique structure, encompassing personal, communal, environmental, and transcendental dimensions. Table 1 provides a list of the 20 items in the SWBQ. In the SWBQ, the personal domain examines how individuals relate to themselves in terms of meaning, purpose, and values in life. The communal domain reflects the quality and depth of interpersonal relationships between the self and others and encompasses concepts such as love, justice, hope, and faith in humanity. The environmental domain concerns care and nurturing for the physical and biological world, incorporating a sense of awe, wonder, and unity with the environment. The transcendental domain explores the relationship between the self and an entity beyond the human level, such as a cosmic force, transcendent reality, or God, involving faith, adoration, and worship directed towards the ultimate source of mystery in the universe. However, three of the five items in the subscale for thid domain refer to "God", one to "creator", and one concerns "prayer", which is also meant as a conversation with God. So, this domain actually measures religiosity and not spirituality. Notwithstanding this, overall, spirituality in the context of the SWBQ can be understood as a state that reflects positive feelings, behaviors, and cognitions with relationships with oneself, others, the transcendent, and the environment. Spiritual well-being, in turn, refers to the ways in which these connections foster a sense of purpose, peace, and fulfillment.

**Table 1:** Items in the SWBQ for the different domains

Personal	Communal
Developing a sense of identity	Developing a love for other people
Developing self-awareness	Developing forgiveness for other people
Developing joy in life	Developing trust between individuals
Developing inner peace	Developing respect for others
Developing meaning in life	Developing kindness towards other people
Environmental	Transcendental
Developing connection with nature	Developing a personal relationship with God
Developing awe at breathtaking view	Developing worship of the Creator
Developing oneness with nature	Developing oneness with God
Developing harmony with the environment	Developing peace with God
Developing a sense of ‘magic’ in the environment	Developing prayer life

For each item, respondents are asked to indicate how they feel the statements describe their personal experience over the last 6 months, using a five-point Likert scale, ranging from very low (rated 1) to very high (rated 5)

The SWBQ is included in a larger questionnaire, the Spiritual Health and Life-Orientation Measure (SHALOM; Fisher, 2010). In addition to the SWBQ, which measures "lived experience", there is a second scale of 20 items, in which participants are asked to indicate what they perceive as the ideal levels for each item (the "ideals" subscale). Fisher (2010) has argued that, since there is no universal standard for determining an “average” level of spiritual well-being in a population, discrepancies between ideal and lived experience, reflecting spiritual dissonance, offer a more useful and meaningful measure of spirituality across different domains. However, aside from a general description of ideals and lived experience components of spirituality, there has been no comprehensive theoretical articulation of what spiritual dissonance actually entail. This contrasts with self-discrepancy theory (Higgins, 1987), which has a well-established framework (Boldero & Francis, 1999) for defining discrepancies between a person’s actual or real self (analogous to the lived experience spirituality component) and their ideal self (analogous to the ideals spirituality component).

Nevertheless, a recent study using SHALOM discrepancy scores found that scores for the personal, communal, and transcendental domains were positively associated with psychological and social quality of life, while the environmental domain was associated with social quality of life (Kuang et al., 2023). However, in that study, the separate scores for the ideals and lived experience were also associated with psychological and social quality of life, thereby, making it difficult to interpret the meaning of the findings of the discrepancy scores. To date, no other published study has examined how general well-being is associated with the SWBQ spiritual well-being domains using the discrepancy/spiritual dissonance approach. This indicates that, in addition to the absence of a sound theoretical foundation, empirical support for the use of discrepancy scores for the SWBQ remains questionable. Given this, the commentary focuses exclusively on the SWBQ.

In a 2018 paper on the SWBQ, the authors stated that the SWBQ had been translated into 27 languages and has been used in over 500 studies internationally (Abhari et al., 2018). Based on a review of ten questionnaires measuring spirituality as a universal human experience, de Jager Meezenbroek et al. (2012) concluded that the SWBQ was the only measure with proven validity and reliability. A recent review by Fisher (2021), published in this journal, analyzed 60 studies worldwide to evaluate the validity and utility of the SHALOM (and, by extension, the SWBQ), and concluded that the SHALOM has demonstrated wide utility and good validity.

A major issue when examining the associations between spiritual well-being and general well-being is tautology (Bambling, 2024). In this context, tautology refers to substantial overlaps in the content of items within spirituality and well-being questionnaires, which may result in measuring the same construct, thereby producing inaccurate and meaningless findings (Bambling, 2024; de Jager Meezenbroek et al., 2012; Garssen et al., 2016). In relation to this, de Jager Meezenbroek et al. (2012) noted that, since only two of its 20 items overlap with general well-being, the SWBQ can be considered unaffected by tautology. Notwithstanding this, more recently, Koenig and Carey (2024), have noted that the SWBQ items tap mainly outcomes of spirituality, not spirituality itself, and that it is confounded by tautology if the four subscales are combined. According to them, only the transcendental domain is relevant to spirituality. Taken together with de Jager Meezenbroek et al.’s (2012) view of the SWBQ, it can be assumed that the separate domain scales (but not the total score) of the SWBQ can be regarded as highly suitable for studying the associations between spirituality and general well-being.

Although Fisher’s (2021) extensive review concluded that the SWBQ has wide utility and good validity, a closer examination of this paper reveals that, while it demonstrated the wide use of the SWBQ, it did not actually cover issues of validity. While the tables (Tables 1 and 2) in that paper included “Validation” columns, the columns simply listed the major statistical procedure used in the different studies, and not the findings supporting validity. Given this limitation, and considering that SWBQ is widely used and has potential for understanding general well-being, it is essential to reassess the value of the SWBQ for well-being research.

**Table 2:** Summary of findings in past studies examining the associations of the SWBQ domains with other well-being areas

Study		#	Nonspiritual well-being		SWBQ domains			
					P	C	E	T
*Correlation Findings (Pearson’s correlation)*								
Abhari et al. (2018)		170	General health	General Health Questionnaire	L _(-.24*)_	T _(-.02) ns_	T _(-.09) ns_	T _(-.07) ns_
			Happiness	Oxford Happiness Scale	M _(.30*)_	L _(.18) ns_	L _(.29*)_	L _(.18) ns_
Elhai et al. (2018)		331	Satisfaction with life	Carmel and Mutran (1997)	M _(.44*)_	M _(.37*)_	L _(.22*)_	T _(.08) ns_
			Will to live	Carmel (2001)	L _(.28*)_	L _(.15*)_	L _(.13*)_	L _(.14*)_
			Perception of health	Single item	L _(.21*)_	T _(.07) ns_	T _(.07) ns_	T _(.09_) _ns_
			Relative perception health	Single item	L _(.24*)_	T _(.07) ns_	L _(.16*)_	T _(.09) ns_
			Depression	Geriatric Depression Scale	M _(-.42*)_	L _(-.28*)_	L _(-.14*)_	L _(-.10) ns_
			Fear of dying	Carmel (2001)	T _(-.09) ns_	T _(-.09) ns_	T _(-.03) ns_	L _(-.24*)_
Gomez & Fisher (2023)		456	Happiness	Oxford Happiness Scale	M _(.33*)_	M _(.34*)_	L _(.15*)_	T _(.08) ns_
Gomez and Watson (2023)^a^		227	Satisfaction with life	Satisfaction with Life Scale	L _(.10) ns_	L _(.28*)_	T _(.07) ns_	Nr
			Loneliness	UCLA Loneliness Scale-Version 3	L _(-.10) ns_	M _(-.43*)_	T _(.04) ns_	Nr
			Self-esteem	Rosenberg Self-Esteem Scale	L _(.21) ns_	T _(.00) ns_	L _(.15) ns_	Nr
Holder et al. (2010)		320	Happiness	Oxford Happiness Scale	M _(.48*)_	M _(.44*)_	L _(.28*)_	L_(.16*)_
			Subjective happiness	Subjective Happiness Scale	M _(.38*)_	M _(.34*)_	L _(.14*)_	L _(.10) ns_
			Single happiness item	Faces Scale (Own Face)	M _(.44*)_	M _(.45*)_	L _(.21*)_	L _(.12*)_
Nunes et al. (2018)		436	Positive relations	Psychological Well-being Scale	L _(.14*)_	L _(.16*_)	T _(.08) ns_	L _(.11*)_
			Autonomy	Psychological Well-being Scale	L _(.22*)_	T _(.09) ns_	L _(.15*)_	L _(.10 ns)_
			Environmental mastery	Psychological Well-being Scale	L _(.25*)_	L _(.15*)_	L _(.18*)_	L _(.17*)_
			Personal growth	Psychological Well-being Scale	L _(.18*)_	L _(.15*)_	L (._13*)_	T _(.09) ns_
			Purpose in life	Psychological Well-being Scale	L _(.23*)_	L _(.15*)_	L _(.13*_)	L _(.14*)_
			Self-acceptance	Psychological Well-being Scale	M _(.30*)_	L _(.16*)_	L _(.14*)_	L _(.10*)_
Rowold (2011) study 1	207		Mental life quality	Developed for study	M _(.37*)_	L _(.24*)_	M _(.31*)_	L _(.20*)_
			Physical life quality	Developed for study	M _(.49*)_	M _(.32*)_	M _(.38*)_	L _(.23*)_
			Emotional life quality	Developed for study	L _(.20*)_	L _(.27*)_	L _(.19*)_	L _(.20*)_
Rowold (2011) study 2	49		Happiness	Oxford Happiness Scale	M _(.38*)_	L _(.13) ns_	T _(.09) ns_	T _(.00) ns_
			Psychological well-being	SF-12	M _(.47*)_	M _(.41*)_	L _(.13) ns_	L _(.23) ns_
Rowold (2011) study 3	164		psychological well-being	WHOQOL	M _(.31*)_	L _(.16*)_	L _(.16*)_	L _(.24*)_
			Perceived chronic stress	Trier Inventory	M _(.31*)_	L _(-.19*)_	L _(.10) ns_	T _(-.06) ns_
Multiple Regression Analysis Findings (Standardized beta values)								
Gomez and Watson (2023)^a^	227		Satisfaction with life	Satisfaction with Life Scale	nr	nr	nr	T _(-.02) ns_
			Loneliness	UCLA Loneliness Scale-Version 3	nr	nr	nr	L _(.12) ns_
			Self-esteem	Rosenberg Self-Esteem Scale	nr	nr	nr	L _(-.10) ns_
Holder et al. (2010)	320		Happiness	Oxford Happiness Scale	M _(.32*_)	L (.25*)	T _(-.01) ns_	L _(-.14) ns_
			Subjective happiness	Subjective Happiness Scale	M _(.34*)_	L _(.21*)_	L _(-.11) ns_	L _(-.16) ns_
			Single happiness item	Faces Scale (Own Face)	M _(.30*)_	M _(.32*)_	L _(-.10) ns_	T _(-.09) ns_
Rowold (2011) study 1	49		Happiness	Oxford Happiness Scale	M _(.42*)_	L _(.28*)_	L _(-.15) ns_	T _(.08) ns_
			Psychological well-being	SF-12	M _(.40*)_	T _(.04) ns_	T _(.01) ns_	T _(-.05) ns_
Rowold (2011) study 3	164		Psychological well-being	WHOQOL	L _(28*)_	T _(.05) ns_	T _(-.09) ns_	L _(.21*)_
			Perceived chronic stress	Trier Inventory	M _(-.30*)_	T _(-.09) ns_	T _(.07) ns_	T _(-.02) ns_

*P* Personal, *C* Communal, *E* Environmental, *TR* Transcendental, *T* trivial, *L* Low, *M* medium, *H* and Hugh, *nr* not reported, # number of participant in the study

In the table, effect sizes for correlations and beta coefficients were interpreted using Cohen (1992) guideline: trivial (T) =  < .10, Low (L) = 0.10, medium (M) = 0.30, and Hugh (H) = 0.50, respectively

^a^Transcendental was regressed on all four SWBQ dimensions

All studies except that by Holder et al. (2010) involved adults. The Holder et al. (2010) study involved children

The study by Rowold (2011) was a four-week follow-up study

The aim of this commentary is to provide a clear and critical review of the literature on the associations between the SWBQ spiritual well-being domains and other areas of general well-being. As the lived experience component in the SHALOM is identical to the SWBQ, findings from this component of the SHALOM are also included in this commentary when referring to the SWBQ. However, given the aim of the paper and the point made by Koenig and Carey (2024) that the total SWBQ score is confounded by tautology, the commentary does not include studies that have examined the SWBQ as a unidimensional global construct. The commentary has also excluded studies that have merged the personal and communal domains to a single personal/communal construct (Leung, 2023; Leung & Pong, 2021; Pong, 2022, 2024), as this three-factor structure does not conform with the originally proposed SWBQ, the NICA (1975) framework, or Fisher’s (1998) elaboration of it.

### Review of Existing Studies Examining the Associations of SWBQ Domains with General Well-Being

To date, numerous studies have examined the associations between the domains of the SWBQ with general well-being (Abhari et al., 2018; Elhai et al., 2018; Gomez & Fisher, 2003; Gomez & Watson, 2023; Holder et al., 2010; Nunes et al., 2018; Rowold, 2011). This relates to concurrent validity, i.e., relationship of target construct to external criterion that is expected to be theoretical related to the target construct (DeVellis & Thorpe, 2022). This form of validity delving into the theoretical underpinnings of the constructs, and ensures that the scale not only measures what it purports to, but does so in a manner consistent with established theories (American Educational Research Association, American Psychological Association, and National Council on Measurement in Education, 2014). Although the SWBQ was not developed to measure general well-being validity as such, the authors who developed this measure concluded in their original SWBQ scale development and validation study (Gomez & Fisher, 2003) that “it could have a high degree of relevance for those interested in research on the interrelations between spiritual life experience and well-being” constructs (p. 1989). Considering this, it could be argued that as the SWBQ domains are relevant proxy measures of general well-being, albeit spirituality, and therefore the theoretical expectation could be that they would show associations with other general well-being measures.

In the context of the SWBQ, concurrent validity in past studies has been analyzed using correlation analysis and multiple regression analysis. When using correlation coefficients, the relationships between each of the four SWBQ aspects and the various well-being measures are determined separately. In the studies that used multiple regression analysis, all four SWBQ aspects were included as predictors for well-being in one analysis. This determines the explained variance in the well-being outcome measure of one SWBQ predictor on top of the variance already explained by the other three SWBQ predictors. However, it is worth noting that this methodology is still unacceptable in instances where the spiritual measure is contaminated by general well-being items (Koenig & Carey, 2025). This is unlikely to be relevant for the SWBQ since only two of its 20 items overlap with general well-being, it can be considered to be have minimal contamination by general well-being items (de Jager Meezenbroek et al., 2012).

To facilitate a clear overview of the studies in this area, Table 2 summarizes their findings, distinguishing between correlational and multiple regression studies. This table presents the reported correlations and includes effect sizes interpretations, based on Cohen’s (1992) guideline for correlation/regression coefficient effect sizes: values of 0.10, 0.30, and 0.50 are small, medium, and large effect sizes, respectively. Considering this, values below 0.10 are considered trivial. All studies, except for Holder et al. (2010) involved adults. The Holder et al. (2010) study involved children. Additionally, except for Rowold (2011; Studies 2 and 3), all other studies were cross-sectional. Study 2 and 3 in Rowold (2011) examined longitudinal data with a four-week interval. Regarding general well-being measures, these studies have examined both hedonic/subjective well-being (i.e., one’s cognitive and affective evaluation of life, including life satisfaction, high positive affect, and low negative affect; Diener, 1984) and eudemonic/psychological well-being (i.e., the outcome of positive goal pursuits, including autonomy, environmental mastery, personal growth, positive relations with others, purpose in life, and self-acceptance; Ryff, 1989) areas.

### Review of Studies Involving Correlational Findings

Several studies have investigated the correlations between the domains of the SWBQ and general well-being (Abhari et al., 2018; Elhai et al., 2018; Gomez & Fisher, 2003; Gomez & Watson, 2023; Holder et al., 2010; Nunes et al., 2018; Rowold, 2011). Overall, the correlation findings revealed positive associations with various general well-being areas, in the theoretically expected directions, but with notable differences across domains.

As shown in Table 2, the associations have been generally more pervasive, consistent and stronger for the personal domain, with at least positive medium effect size associations with happiness (Abhari et al., 2018; Gomez & Fisher, 2003; Holder et al., 2010; Rowold, 2011), satisfaction with life (Elhai et al., 2018), quality of life (Rowold, 2011), psychological well-being (Rowold, 2011), and negative medium effect size associations with depression (Elhai et al., 2018), and chronic stress (Rowold, 2011) in a total of 24 references in 28 studies referenced here.

The relations of the communal domain with general well-being areas have been less often found and inconsistent inconsistent among 20 references in the 28 studies reported here. There have been positive medium effect size associations with satisfaction with life (Elhai et al., 2018), happiness (Gomez & Fisher, 2003; Holder et al., 2010; Rowold, 2011), psychological well-being (Nunes et al., 2018; Rowold, 2011); and physical life quality (Rowold, 2011). For the environmental domain, associations with general well-being have been rare, with only one study reporting positive medium effect size association with mental life quality and physical life quality (Rowold, 2011). The associations for the transcendental domain have been rare, with virtually all associations when found being of trivial or low effect sizes.

Overall, still in 11 references in 28 studies reported here, there appears to be more significant correlations for the personal domain and to a lesser degree the communal domain with general well-being, but less so for environmental and transcendental domains.

### Review of Studies Involving Multiple Regression Analysis Findings

Only a handful of studies have used multiple regression analysis to examine the unique associations of SWBQ domains with general well-being (Gomez & Watson, 2023; Holder et al., 2010; Rowold, 2011) in a total of 18 references in 28 studies reported here. As shown in Table 2, generally the personal domain was associated significantly with various general well-being areas, with medium effect sizes, especially with hope, life satisfaction and personal well-being (Gomez & Watson, 2023), happiness (Holder et al., 2010), and psychological well-being (Rowold, 2011); and negative association with chronic stress (Rowold, 2011). The associations for the communal domain with general well-being areas have been less often found and more inconsistent and weaker. There has been positive medium effect size association with happiness (Holder et al., 2010). For both the environmental and transcendental dimension, the associations with other general well-being measures were generally not as significant. It is worth noting that although the study by Rowold (2011) was a longitudinal study, it involved a four-week interval. Given that the spiritual well-being domains are trait-like measures that develop over extended periods, caution must be therefore exercised when these findings are interpreted in causal terms. Overall, therefore, the findings involving multiple regression analysis indicate by far noteworthy significant unique association for only the personal domain with general well-being.

## Conclusions

Given the lack of empirical evidence linking the communal, environmental and transcendental domains with general well-being, especially among adults, it would appear that in general, except for the personal domain in the SWBQ, all the other domains may *have less* associations with most aspects of general well-being areas in this group..Consequently, it can be speculated that the personal domain can be considered *to have most relevance* for general well-being. Considering this, it can be argued that despite endorsement of the SWBQ as a useful measure of spiritual well-being (de Jager Meezenbroek et al., 2012), it would be prudent to excersise some caution when viewing the SWBQ domains in well-being terms (Gomez & Watson, 2023), whether they pertain to subjective/hedonic or eudemonic/psychological well-being.

Our findings in this commentary also raises broader concerns about the spiritual dimensions proposed by the NICA and Fisher’s multidimensional model of spiritual well-being (Gomez & Watson, 2023). The meta-analysis by Garssen et al. (2021) that involved 48 longitudinal studies reported that although there was a significant positive association between religion or spirituality and with mental health (distress measure, life satisfaction, well-being, and quality of life), this was of small effect size [r = .08 (95% CI 0.06–0.10)]. In line with this, several researchers have suggested that spirituality is not inherently relevant to general well-being (Eichhorn, 2011; Garssen et al., 2021; Leondari & Gialamas, 2009; Prati, 2024). Considering this, an alternative explanation for the lack of associations between the SWBQ domains and general well-being is that such relationships are weak and not always evident.

Overall, while it is concluded that that apart from the personal domain,the other SWBQ domains show weak or questionable associations with general well-being, further research is needed for a comprehensive understanding of spiritual well-being’s connections to other dimensions of well-being. In particular, longitudinal research using alternative theoretical models (comprehensively reviewed by Carroll, 2001; Rovers & Kocum, 2010) and/or alternative measures of spirituality and spiritual well-being (de Jager Meezenbroek et al., 2012; Kapuscinski et al., 2010; Lun & Bond, 2013; Monod et al., 2011) that are more culturally (Lun & Bound, 2013) and religiously sensitive (e.g., religious beliefs within a specific faith tradition; Ahmad & Khan, 2016; Prati, 2024), as well as consideration of gender, sexual orientation, and developmental factors (Huffman et al., 2020; Oláh & Koronczai, 2021; Vosloo et al., 2009) will be valuable as they would provide deeper insight into how spirituality interacts with well-being across different populations. Relatedly, it is important that such studies control for tautology to provide meaningful results, and/or consider statistical methodology well suited to handle tautological issues in research on the relationships between spirituality and general well-being (Koenig & Carey, 2025).

## Data Availability

Not applicable.
